# Trends in Reproductive Indicators of Green and Hawksbill Sea Turtles over a 30-Year Monitoring Period in the Southern Gulf of Mexico and Their Conservation Implications

**DOI:** 10.3390/ani12233280

**Published:** 2022-11-24

**Authors:** Melania C. López-Castro, Eduardo Cuevas, Vicente Guzmán Hernández, Ángeles Raymundo Sánchez, Rosa C. Martínez-Portugal, Diana J. Lira Reyes, Jorge Ángel Berzunza Chio

**Affiliations:** 1 Pronatura Península de Yucatán, A.C., Programa para la Conservación de la Tortuga Marina, Merida 97205, Yucatán, Mexico; 2 Departamento de Recursos del Mar, Consejo Nacional de Ciencia y Tecnología—Centro de Investigación y de Estudios Avanzados del Instituto Politécnico Nacional Unidad Mérida, Merida 97310, Yucatán, Mexico; 3 Comisión Nacional de Áreas Naturales Protegidas, Área de Protección de Flora y Fauna Laguna de Términos, Ciudad del Carmen 24129, Campeche, Mexico; 4 Département des Sciences du Bois et de la Forêt, Faculté de Foresterie et de Géomatique, Université Laval, Quebec, QC G1V 0A6, Canada; 5 Comisión Nacional de Áreas Naturales Protegidas, Parque Nacional Sistema Arrecifal Veracruzano, Veracruz 91919, Veracruz, Mexico; 6 Secretaría de Medio Ambiente, Biodiversidad, Cambio Climático y Energía de Campeche, Subdirección de Vida Silvestre, San Francisco de Campeche 24095, Campeche, Mexico

**Keywords:** long-term monitoring, population trends, reproductive parameters, hawksbill turtle, green turtle

## Abstract

**Simple Summary:**

From 1990 to 2021, the number of hawksbill and green turtle nests has prominently increased thanks to the long-term protection of primary nesting sites in the Southern Gulf of México. However, despite not finding a statistical significance in the temporal trends, the size of nesting females, the clutch size, hatching, and emergence success are slowly decreasing in response to multiple conditions. Our study suggests that the protection efforts at nesting beaches show promising results. However, other ongoing natural and anthropic drivers are acting on sea turtle populations and their habitats and could dampen their recovery by reducing their reproductive output and hatchling production. Thus, because sea turtles are highly migratory animals moving between different countries, aside from protecting key nesting sites, restoring and monitoring crucial foraging habitats should be an immediate priority requiring international cooperation.

**Abstract:**

Long-term monitoring programs of species at risk are efficacious tools to assess population changes, evaluate conservation strategies, and improve management practices to ensure populations reach levels at which they can fulfill their ecological roles. For sea turtles, annual nesting beach surveys are the most accessible method to estimating the population abundance and reproductive output, especially when these are done in primary nesting sites. However, little data exist on the long-term assessment of these parameters. Here, we present the trends of the nest abundance, female size, hatching, and emergence success of hawksbill (*Eretmochelys imbricata*) and green (*Chelonia mydas*) turtles at key nesting beaches in the southern Gulf of Mexico over 31 years (from 1990 to 2021). The nest abundance showed an increasing trend in both species as a result of the sustained protection and conservation effort, but there was no significant temporal trend in the annual female size, clutch size, hatching, and emergence success. However, these indicators showed decreasing mean values over the last decade and should be closely monitored. We suggest these decreases link to the combined effects of ocean warming and anthropogenic pressures affecting the sea turtle foraging grounds. Aside from protecting key nesting sites, protecting and restoring crucial foraging habitats should be an immediate priority requiring international cooperation.

## 1. Introduction

The recovery of species at risk, particularly those with a long-life span such as sea turtles, requires long-term monitoring programs to evaluate and standardize conservation and management methods, understand the current population trends, and ensure that populations reach levels at which they can fulfill their ecological functions in the ecosystems they inhabit [1,2,3,4]. In large marine organisms, assessing changes in the population abundance is a very difficult task because of their wide distribution, migratory nature, and restricted access to different age classes [5]. Therefore, for any given age class, selecting and measuring the temporal variability of proper indicators can give information on the population status [6].

Most of the population assessments of sea turtles to date are based on the monitoring of the nesting trends [7,8] and other demographic indicators such as the female size and survival probability [6,9,10], clutch frequency, and remigration intervals [5] of rookeries around the world. However, other indicators such as the clutch size, hatching, and emergence success can also detect changes in the reproductive output of the populations [8,11] and give insights into the population’s health and the potential for recovery.

In addition to the nest abundance, female size, clutch size, hatching, and emergence success are used by international organizations such as the International Union for the Conservation of Nature (IUCN) to evaluate the species’ conservation status and dictate their category of risk at regional and global levels [12]. However, the accurate categorization of the conservation status requires updated information from as many rookeries and foraging areas as possible within each regional management unit. To this end, long-term monitoring programs play an important role as information providers.

Rookery assessments demand a strict technical robustness and reliability. Ideally, index nesting beaches should be under the same management, with uninterrupted monitoring for more than ten years, have yearly trained personnel in charge, and have open access reports of the recorded data [5,13,14]. The long-term data obtained with the highest quality control standards are crucial for assessing the recovery of sea turtle populations and serve as technical platforms for defining the adaptive management strategies potentially replicable at other nesting beaches.

In México, sea turtle monitoring has been conducted for over 50 years [15], which provided information on the reproductive biology of the six species nesting in the Mexican Pacific and Atlantic coasts. However, few publications analyze the long-term tendencies of the reproductive parameters as an update on the rookeries’ condition status and how sea turtle conservation programs perform. For this reason, the goals of this study were (i) to evaluate the long-term status of the reproductive parameters, including the nest abundance of hawksbill (*Eretmochelys imbricata*) and green (*Chelonia mydas*) sea turtles at seven nesting beaches of the southern Gulf of Mexico, and (ii) to highlight the significance of systematic long-term monitoring protocols to detect population changes and evaluate the recovery of these species in the southern Gulf of Mexico.

The southern Gulf of México is a strategic conservation area for several sea turtle species and their populations. This region is home to the largest hawksbill nesting population in the West Atlantic and the seventh in the world, representing 25% of all documented nests in the Wider Caribbean [16,17]. High nest densities of green sea turtles also occur here, considered to be one of the four largest rookeries in the Caribbean [18], with an average 11.6% increase in nest abundance per year [19]. Only 34 sites within the Wider Caribbean report more than 1000 nests per year, and 15 of them are in the southern Gulf of Mexico [16]. The protection and biological monitoring of the sea turtle nesting in this region started in the 1990s and continues until today. Previous studies show that the annual average number of nests recorded at these nesting beaches has slowly increased over three decades [20,21]. Thus, evaluating the temporal and spatial variation of the reproductive parameters and nest abundance at these seven nesting beaches will provide updated insightful information on the recovery trend of these species in the southern Gulf of Mexico.

## 2. Materials and Methods

### 2.1. Data Collection

**Figure 1:**
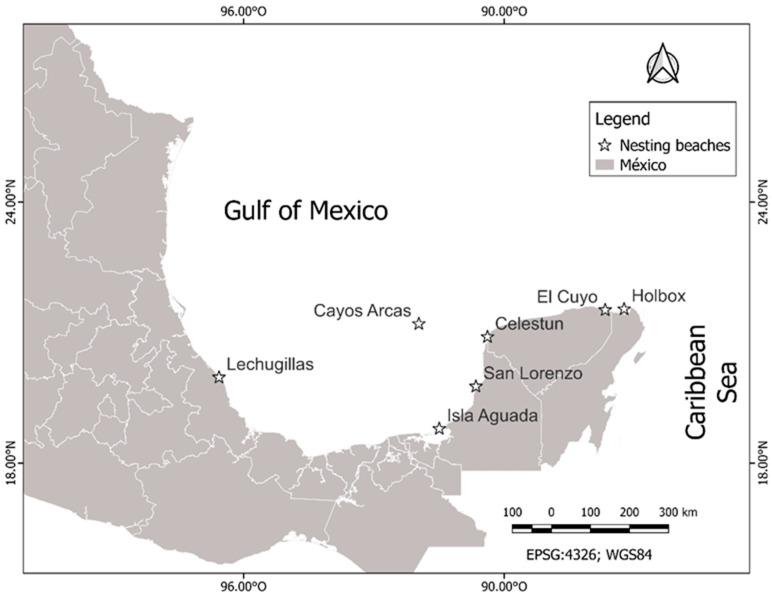
Location of the seven nesting beaches along the Gulf of Mexico coast where the study was conducted.

For this study, we considered the monitoring data of hawksbill and green sea turtle nesting from 1990 to 2021 at seven nesting beaches in the southern Gulf of Mexico: Lechuguillas in Veracruz; Isla Aguada, Cayos Arcas, and San Lorenzo in Campeche; Celestún and El Cuyo in Yucatán; and Holbox in Quintana Roo (Figure 1). All of the nesting beaches implemented a standardized protocol for the monitoring and collection of the nesting data. Although the records of nest numbers exist for the extent of the study period at Isla Aguada, Celestún, El Cuyo, and Holbox, the records at Cayos Arcas start in 2002, and 1994 at San Lorenzo and Lechuguillas. Data on the female size, clutch size, hatching, and emergence success were only available from 1995 to 2021 in Lechuguillas, Celestún, and El Cuyo, from 2002 in Cayos Arcas, and 2003 in San Lorenzo.

In general, monitoring surveys occurred three or more times per week between April and October (the duration of the nesting season), thus observing the minimum standards of a B protocol, level 1 [22]. The nesting monitoring consisted of night beach surveys on foot or using motorized vehicles (ATVs). During these surveys, females and their nests were recorded, including geographic coordinates of their location and the position on the beach profile, and the time and date of the encounter. The nests laid near the tide line or in areas of imminent risk [23] were relocated to safer areas of the beach or hatcheries. The females were measured using flexible tapes to obtain the curved carapace length (standard and minimum) [24] and checked for flipper tags.

The average clutch size, hatching, and emergence success were estimated based on the analysis of the nest contents after the emergence date [25]. Beginning in 2010, during each nesting season, a minimum of 25% of the total number of undisturbed hawksbill nests and at least 15% of green turtle nests were examined at Celestún, El Cuyo, and Holbox. These percentages are representative of the total number of recorded nests [26]. In Lechuguillas, over the 28 green turtle nesting seasons, on average 39% of the in situ nests were analyzed. This percentage ranged from 0.1% in 1994 (almost all nests were relocated to hatcheries) to 70% in 2009. Hawksbill turtle nests were relocated or incubated in containers, except for 2 nests kept in situ in 2018. In Isla Aguada, before 2013, 80% of green and between 80 and 100% of hawksbill nests were analyzed. After that, at least 25% of green and more than 80% of hawksbill nests were analyzed annually. The percentage of analyzed nests in San Lorenzo was 90% or higher and between 50 and 100% in Cayos Arcas.

### 2.2. Data Preprocessing

The databases contained more than 100,000 entries of females and nests recorded over 31 years. Because we were interested in evaluating the changes in four reproductive parameters (the female size, clutch size, hatching, and emergence success), we conducted an exhaustive data quality control and eliminated those entries with incomplete or missing data before running the analysis. The records of the nests that did not have information on the clutch size, hatching, or emergence success were excluded from the analysis, as well as records of females that did not have complete size information. We ended with a total of 70,001 nest observations and 20,925 female observations (90,926 total) to evaluate changes through time within and between nesting sites. For this same reason, although all the nesting beaches had a complete time series record on the nest abundance, some had a small sample size or did not have at least 10 years of continuous records of the other variables, and thus were excluded from the analysis. This was the case for the hawksbill turtle data from Lechuguillas (incipient nesting) and San Lorenzo (incomplete time series), and for the size data of green turtles from Celestún (incipient nesting) and Cayos Arcas (incomplete time series), however, their statistical summary is included as a reference.

### 2.3. Trends of Nesting Abundance

The nests of both sea turtle species were recorded each year from April to October and even November when the nest density was high, which is the duration of the nesting season. Daily surveys were conducted for 27 days each month unless the weather conditions during major storms were unsuitable for the safety of our personnel. The beaches were surveyed twice every night either on foot or using ATVs. To keep track of the nests, each new nest was marked with a successive number during the night surveys. When the hatching season started, the unmarked nests were also recorded, increasing the accuracy of the nesting abundance estimates.

### 2.4. Carapace Length Distributions

Because some females were observed and measured more than once in the same nesting season, only the first record was included in the numerical analysis. Over the 31 years of the data collection, the protocols for measuring the hawksbill turtles changed. At first, the standard curved carapace length (CCLstd) was recorded (measured from the midline point of the nuchal scute to the tip of the largest 12th marginal). However, because these marginal scutes can often break, the measure changed to the minimum curved carapace length (CCLmin). This is measured from the midline point of the nuchal scute to the notch of the two marginal scutes, giving a more precise measure of the size of the turtle. To make the data comparable, we did a correlation analysis between CCLstd and CCLmin for this species, followed by a fitting linear model using the package STATS in R [27]. This gave us the equation parameters to transform CCLstd to CCLmin (LCCmin = 8.7577632 + (0.8657161 × LCCstd)). At Lechuguillas, starting in 2019, the minimum straight carapace length (SCLmin) was measured instead of CCLmin. In this case, SCLmin was transformed to CCLmin using the formula LCCmin = 0.028 + (1.051 × SCL) suggested in another study [28].

While the measurement protocols for nesting individuals have evolved over the thirty years of this study, the personnel in charge of the biological monitoring at these index beaches receives annual technical training to comply with the internationally recognized standards [5,13], and most of them have more than 3 years of professional experience (some more than 15 years).

### 2.5. Clutch Size, Hatching, and Emergence Success

The clutch size (CS) was determined in two different ways: by counting the number of eggs laid by the females when the nests were relocated or by analyzing the residual contents of nests after the emergence of the hatchlings. This analysis consisted in classifying and counting the egg shells (S), undeveloped eggs (UD), unhatched eggs (UH), pipped eggs with live (PL) and dead hatchlings (PD), and live (L) and dead (D) hatchlings (inside the nest). Only shells that made up more than 50% of the egg size were counted [25] to estimate the clutch size. We used distribution quantiles to define the minimum and maximum values of the clutch size to eliminate the outliers associated with the documentation of partial clutches and the possibility of two adjacent nests being counted as a single clutch during the excavation [29]. As a result, we eliminated hawksbill nests with ≤32 eggs or ≥194, and in the case of green turtle nests, we excluded those with ≤53 or ≥154.

The information obtained from the undisturbed in situ nest contents was used to estimate the hatching success (turtles that hatch out of their egg) and emergence success (turtles that successfully crawled out of the nest), based on the formulas [25]:

Hatching success (%) = (S ÷ CS),(1)

where: CS = (S − (L + D)) + UD + UH + PL + PD,(2)

Emergence success (%) = (S − (L+D)) ÷ CS(3)

Because our main goal was to evaluate the reproductive health of the nesting populations, only undisturbed nests were included in the hatching and emergence success assessment.

### 2.6. Statistical Analysis

We conducted a multivariate normality test using the package MVN in R [30] to test the normal distribution of the data; this was not the case except for the size of the females. Additionally, the variables measured were not statistically independent. We used a generalized additive models (GAM) approach to evaluate the trends of nests in the seven assessed beaches. To account for an autocorrelation when evaluating the temporal effect (season) on the annual counts of nests (the number of nests) during 1995 and 2021, we used a negative binomial link function. We used a first-order autoregressive process to account for the autocorrelation to evaluate the temporal effect on the following reproductive parameters: the clutch size, emergence success, hatch success, and minimum curved carapace length (CCLmin). The models were fit using thin-plate regression splines to evaluate the effects of nonlinear covariates, and the smoothness parameters were obtained using the REML. The models were fitted using the gam function in the mgcv R package [27,31]. In the cases where the GAMs did not find a significant temporal effect on the response variable, we present the time series plots (mean ± SD) of the response variable.

## 3. Results

### 3.1. Trends in Nest Abundance

Over the study period, the annual number of hawksbill nests has changed one order of magnitude in Celestún, El Cuyo, Isla Aguada, and San Lorenzo, and two orders of magnitude in Holbox in the last decade. Hawksbill nesting at Lechuguillas is incipient. In green turtles, the nest abundance has changed three orders of magnitude in Lechuguillas, two in El Cuyo and Isla Aguada, and only one in Holbox and Cayos Arcas. In Celestún, the first nest was recorded in 2014 and nesting has remained sporadic, but the number of nests has been increasing since 2016 (Supplementary Figure S1).

**Figure 2:**
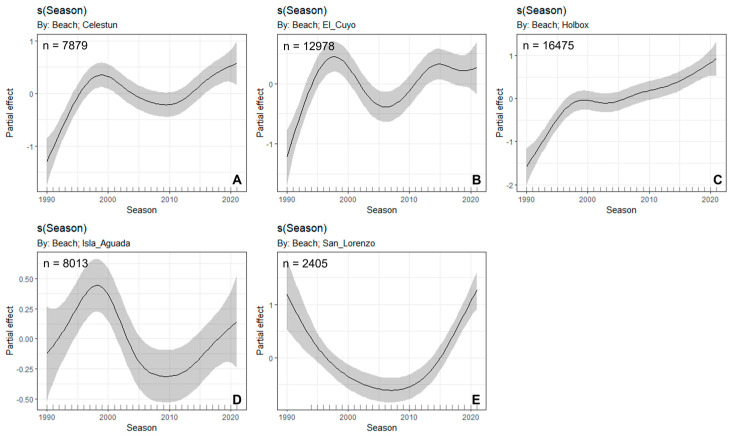
Trends in nest abundance of hawksbill turtles (*Eretmochelys imbricata*) at Celestún (**A**), El Cuyo (**B**), Holbox (**C**), Isla Aguada (**D**), and San Lorenzo (**E**). The black line is the smooth spline line, and the grey areas are the 95% Bayesian confidence intervals. Lechuguillas was excluded from the analysis due to its small sample size, and Cayos Arcas is not a Hawksbill nesting site.

Overall, the number of hawksbill nests increased significantly from 1990 to 2021, except in Isla Aguada and San Lorenzo (Figure 2; *p* < 0.05, R^2^ = 0.814, Table 1 and Table 2). At Celestún and El Cuyo, the number of nests increased from 1990 to 1997, but was followed by a declining trend between 2009 and 2005, respectively. At Holbox, the trend has been increasing since 1990. Isla Aguada had an increasing trend up to 1997, followed by a decline until 2010, and then it has had an increasing trend again. San Lorenzo had a declining trend until 2008, followed by an increasing trend since then.

**Figure 3:**
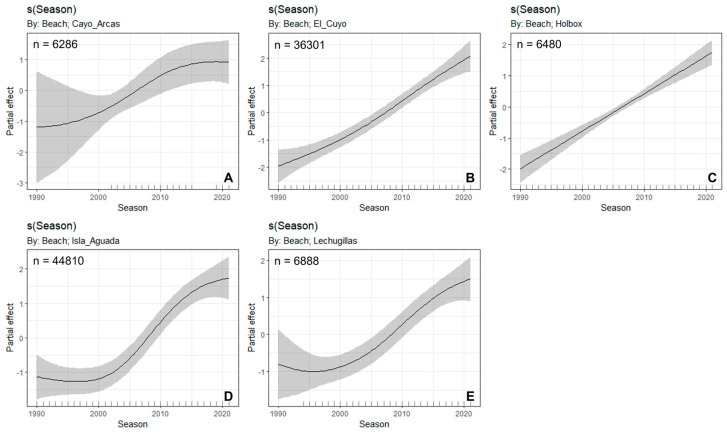
Trends in nest abundance of green turtles (*Chelonia mydas*) at Cayos Arcas (**A**), El Cuyo (**B**), Holbox (**C**), Isla Aguada (**D**), and Lechuguillas (**E**). The black line is the smooth spline line, and the grey areas are the 95% Bayesian confidence intervals. San Lorenzo and Celestún were excluded from the analysis due to their small sample size.

The nesting trend of green turtles presented a biannual pattern characterized by a peak (even years) followed by a low (odd years), from 1990 to 2004 in Cayos Arcas, El Cuyo, Lechuguillas, and Holbox. This pattern reversed from 2005 to 2009 at these sites, with odd years presenting high numbers of nests, but in 2010, the biannual pattern was temporarily disrupted. The number of nests increased each year until 2013 when the biannual pattern was reestablished, suggesting an overlap of the recruiting cohorts. However, in 2019, the pattern broke again with a decrease in the number of nests in Holbox, and an increase in those in Lechuguillas, Isla Aguada, and El Cuyo that continued until 2020; in 2021, the nest numbers decreased again in all the nesting sites (Supplementary Figure S2). However, the number of green turtle nests showed a significant increase from 1990 to 2021 (Figure 3, *p*-value < 0.05, R^2^ = 0.651; Table 3), particularly at Cayos Arcas, El Cuyo, Isla Aguada, and Lechuguillas. Holbox was the only site with a negative trend, although it is not statistically significant (Table 3).

### 3.2. Carapace Length Distributions

**Figure 4:**
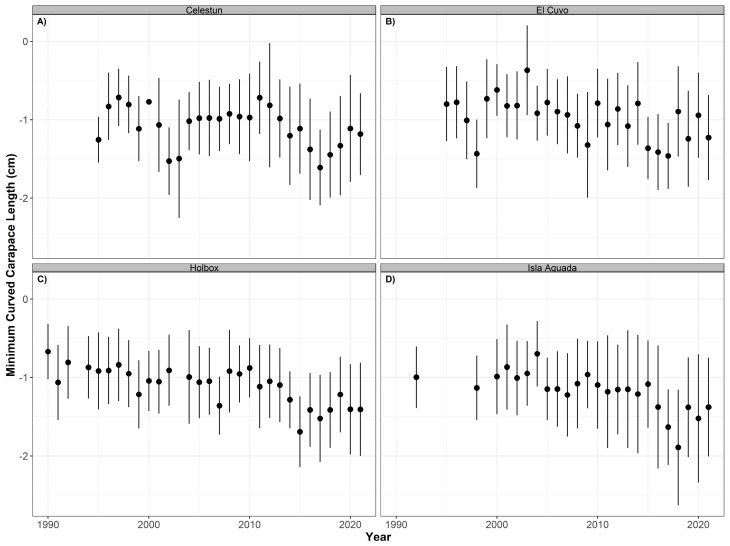
Time series of minimum curved carapace length (annual mean ± SD) for hawksbill turtles (*Eretmochelys imbricata*) nesting at Celestún (**A**), El Cuyo (**B**), Holbox (**C**), and Isla Aguada (**D**) from 1990 to 2021.

**Figure 5:**
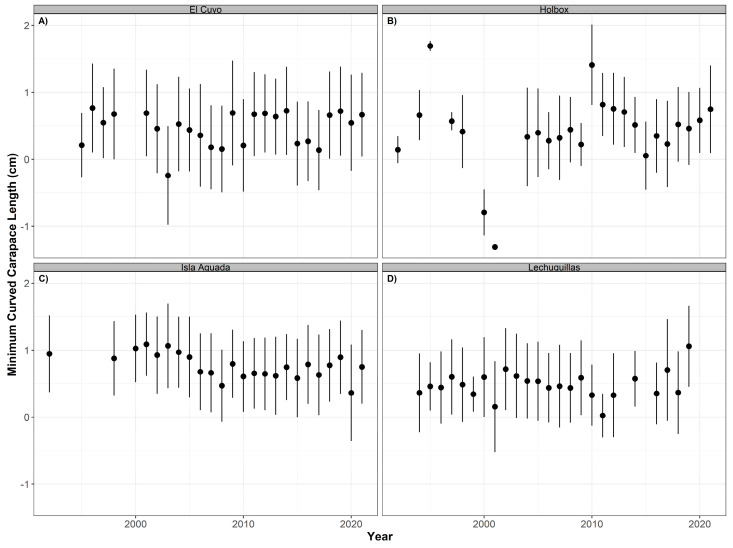
Time series of minimum curved carapace length (annual mean ± SD) for green turtles (*Chelonia mydas*) nesting at El Cuyo (**A**), Holbox (**B**), Isla Aguada (**C**), and Lechuguillas (**D**) from 1990 to 2021.

We did not detect a significant temporal trend for the minimum curved carapace length for either hawksbill (*p* > 0.05, R^2^ = 0.122) or green (*p* > 0.05, R^2^ = 0.044) sea turtles at any of the nesting beaches. Summary statistics are reported in Table 4. However, from 2010 to 2021, the mean size for the hawksbill turtles decreased in Celestún, El Cuyo, Holbox, and Isla Aguada (Figure 4). This is not the case for the mean size of green turtles. The annual mean size increased in Lechuguillas in the same period and remained relatively constant in El Cuyo and Isla Aguada (Figure 5).

### 3.3. Temporal Distribution of Clutch Size, Hatching, and Emergence Success

**Figure 6:**
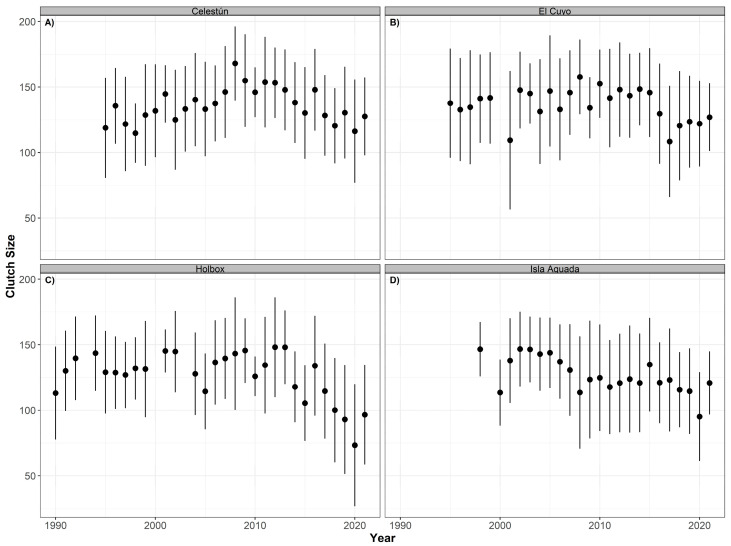
Time series of clutch size (annual mean ± SD) for hawksbill turtles (*Eretmochelys imbricata*) nesting at Celestún (**A**), El Cuyo (**B**), Holbox (**C**), and Isla Aguada (**D**) from 1990 to 2021.

No significant temporal effects were found for the clutch size, hatching, and emergence success for hawksbill or green turtles at any of the nesting beaches, probably caused by the high intra-annual variability of the data (Table 4). For hawksbill turtles, the mean clutch size has fluctuated through the 31 years of study (although with no significant differences: *p* > 0.05, R^2^ = 0.169). In Celestún, the mean clutch size has slowly increased since 1990 and reached a peak in 2008, after which it started to decrease but without reaching its lowest value from 1998. In El Cuyo, the lowest mean value was observed in 2001, then it increased in 2002 and remained stable until it dropped again in 2016 and 2017. Since then, the mean value has remained at approximately 125 eggs, lower than the mean clutch size in the mid-90s. Holbox showed a similar pattern with mean values decreasing since 2014. In Isla Aguada, the mean dropped in 2000 and increased in 2001. It stayed relatively stable until it dropped again in 2008. Despite a slight increase, the mean clutch size dropped again in 2020 and increased in 2021, but the values have not reached those observed in 1998 (Figure 6).

**Figure 7:**
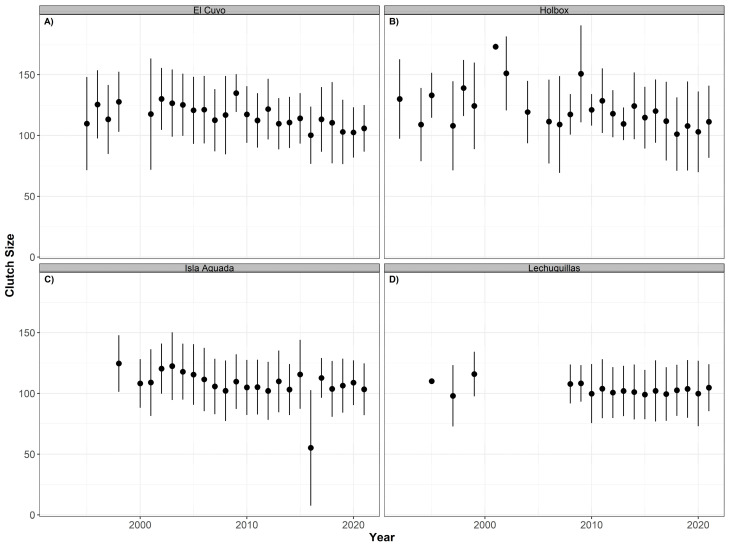
Time series of clutch size (annual mean ± SD) for green turtles (*Chelonia mydas*) nesting at El Cuyo (**A**), Holbox (**B**), Isla Aguada (**C**), and Lechuguillas (**D**) from 1990 to 2021.

No significant trends were detected in green turtles (*p* > 0.05, R^2^ = 0.057) either. The mean clutch size has not fluctuated much in Lechuguillas over the study period. We detected a higher variation in Holbox from 1990 to 2009, and after that, the mean values were closely similar. In El Cuyo and Isla Aguada, the lowest mean values were observed in 2016 (Figure 7).

**Figure 8:**
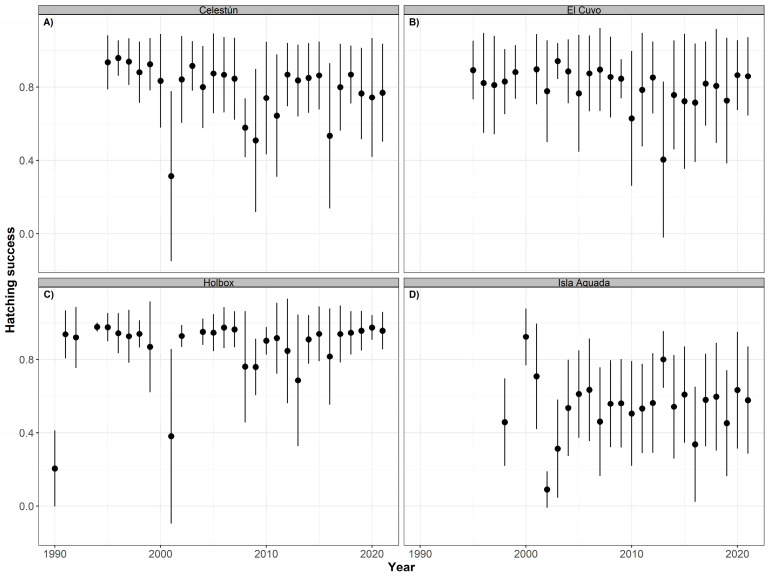
Time series of hatching success (annual mean ± SD) for hawksbill turtles (*Eretmochelys imbricata*) nesting at Celestún (**A**), El Cuyo (**B**), Holbox (**C**), and Isla Aguada (**D**) from 1990 to 2021.

As for the hatching (HS) and emergence (ES) success, we did not detect a significant temporal trend for hawksbill (HS: *p* > 0.05, R^2^ = 0.306 and ES: *p* > 0.05, R^2^ = 0.311) or green (HS: *p* > 0.05, R^2^ = 0.195 and ES: *p* > 0.05, R^2^ = 0.298) turtles in any of the nesting sites. The overall mean hatching success for hawksbill turtles was higher in Holbox (91.72% ± 22.75), followed by Celestún (83.36% ± 25.14), and El Cuyo (80.98% ± 25.9), while it was lower in Isla Aguada (56.24% ± 30.92) (Table 1). The hatching success also varied between the years although the trends were not statistically significant. In most of them, the mean hatching success values were above 60% in all the nesting sites except Isla Aguada. However, low values were documented in 2001 in Celestún and Holbox; in 2013 in El Cuyo; and in 2009 and 2016 again in Celestún (Figure 8).

**Figure 9:**
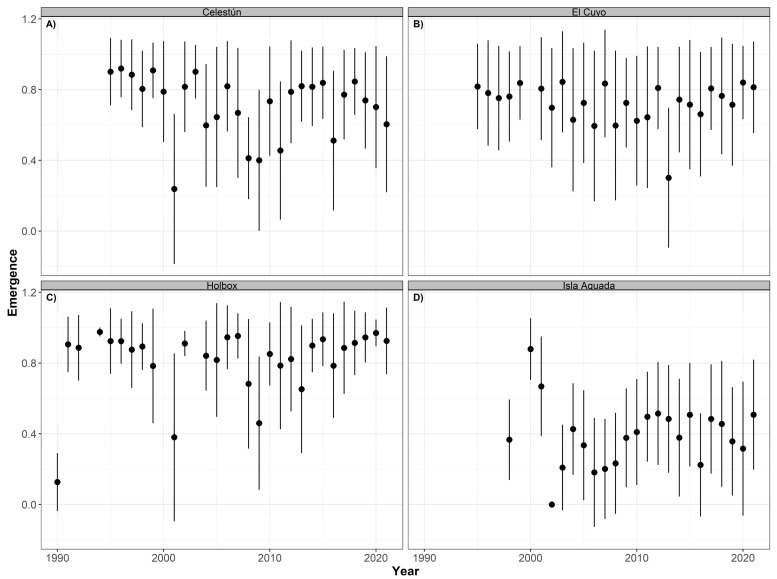
Time series of emergence success (annual mean ± SD) for hawksbill turtles (*Eretmochelys imbricata*) nesting at Celestún (**A**), El Cuyo (**B**), Holbox (**C**), and Isla Aguada (**D**) from 1990 to 2021.

As expected, the years with the lowest emergence success of hawksbill nests coincided with those with the lowest hatching success, particularly in Isla Aguada (Figure 9). However, in addition to those, Celestún and Holbox had more years with a low emergence success than El Cuyo, which is reflected in their annual mean (Table 4). The declines in the hatching and emergence success in these years are probably associated with changes in the environmental conditions of the beach.

**Figure 10:**
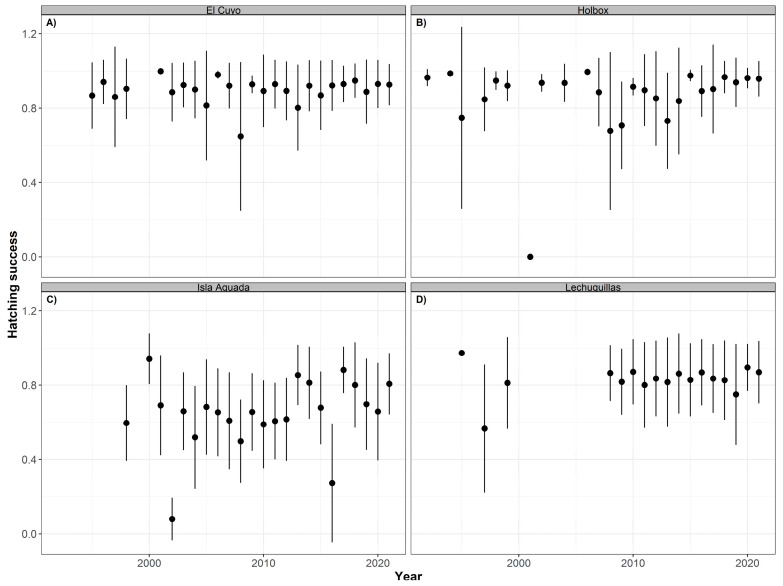
Time series of hatching success (annual mean ± SD) for green turtles (*Chelonia mydas*) nesting at El Cuyo (**A**), Holbox (**B**), Isla Aguada (**C**), and Lechuguillas (**D**) from 1990 to 2021.

**Figure 11:**
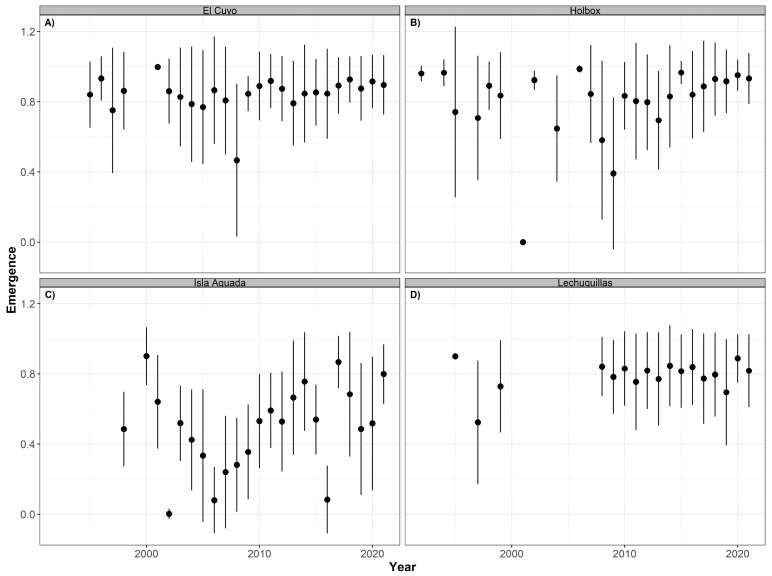
Time series of emergence success (annual mean ± SD) for green turtles (*Chelonia mydas*) nesting at El Cuyo (**A**), Holbox (**B**), Isla Aguada (**C**), and Lechuguillas (**D**) from 1990 to 2021.

Similar non-significant trends were observed in green sea turtles. The overall mean hatching success was highest in Holbox (91.20% ± 17.62), followed by El Cuyo (89.40% ± 16.71), Lechuguillas (84.09% ± 19.81), and Isla Aguada (65.31% ± 26.01) with slightly lower values of mean emergence success (Table 4). The annual mean values of the hatching (Figure 10) and emergence (Figure 11) success were higher in most years, except 2001 in Holbox and 2008 in El Cuyo. Lower mean values were also observed in 2002 and 2016 in Isla Aguada.

## 4. Discussion

Our study is another example of how the long-term protection and monitoring of sea turtle nesting sites can aid in the recovery of sea turtle populations and provide science-based information to guide management and conservation measures. We highlight the relevance of consistency in the spatial and temporal monitoring efforts to contribute scientifically robust information that is critical for improving conservation and management strategies.

### 4.1. Trends in Nest Abundance

The number of nests of hawksbill and green turtles showed a significant increasing trend in almost all of the nesting sites, and this is the result of intensive and technically robust conservation actions carried out in the Gulf of Mexico. This underscores the benefits of sustaining long-term monitoring and protection programs for the recovery of populations [3,5,7,32] and the importance of the southern Gulf of Mexico for the reproduction of sea turtles, especially for hawksbills since the number of nests is decreasing in many nesting sites in the Wider Caribbean Region [16]. Except for Isla Aguada and San Lorenzo, our study sites join the list of the few index nesting sites where the abundance of hawksbill nests is increasing [29]. The mean annual sea surface temperature in the Wider Caribbean has increased over the past decades [33], and climate projections estimate an increase between 1.37 and 2.15 °C in this century. However, the intensity of the increase will be lower in the north of this region [34]. If suitable temperature conditions for the nesting of sea turtles continue to decline in nesting sites in the south of the Caribbean, most of the nesting beaches of the Yucatán Peninsula and the Gulf of Mexico will be key if beach temperatures remain within the optimal thermal range for the incubation and development of hatchlings [35]. Therefore, additional efforts to stop or reduce other anthropogenic threats such as land use change and plastic pollution are needed to safeguard these nesting sites and the integrity of the coastal dune where more than 90% of the nesting occurs. Nevertheless, in addition to the continued evaluation of the reproductive parameters in the coming years, selecting and evaluating habitat health indicators at major foraging sites should be a priority to determine if the current trends are related to the declining conditions of foraging sites, and evaluate the possible solutions to revert them.

### 4.2. Carapace Length Distribution through Time

In the past century, sea turtles were a target species in many fisheries around the world, and their overexploitation drove them to the risk of extinction. Because larger individuals of every species were consistently selected and killed, the mean body size of the reproductive sea turtle populations decreased [36,37], a condition considered a sign of a population under extractive pressure. In México, with the banning of the fisheries in the early 1990 and the consequent recovery of green and hawksbill sea turtles, the mean size of their reproductive populations was expected to increase over time. Yet, we observed a general decreasing trend in the sizes of nesting sea turtle females.

There are some possible explanations for this. The first one is that the populations of green and hawksbill turtles in our region are in a recovery phase of different levels, with green turtles presenting an evident increasing tendency. In the North Atlantic, the estimated age at maturity is between 15 and 25 in hawksbills [38], and 26 years or more for green turtles [32], although the mark-recapture analysis and autografting of this species in the Yucatán peninsula estimates a first nesting age between 14 and 16 years in Quintana Roo [39] and 18 in Campeche [40]. Our data on the nesting trends over the past 31 years show the first peak in the number of nests after approximately 15 years of the beginning of our research on hawksbill turtles, and about 20 years in green turtles. These peaks are related to the level of protection of these beaches in past years and to the recruitment of young adults to the reproductive population, which means not enough time has passed to detect an increment in size, because the proportion of neophytes is still greater than the proportion of larger experienced females. Neophytes have not reached sizes similar to those of old remigrant females, but the gap is being filled, and the size trend will be increasing along with the population stock until it reaches stability.

The population’s growth depends on the recruitment of neophytes. In expanding populations, the proportion of neophytes is expected to increase over time [41] and this is often the case unless, for some unpredicted event (e.g., years with extreme temperatures, drought, and heavy rainfall affecting the incubation environment), the stock expected to recruit is completely or partially lost. It is assumed that the size of the females in a recovering population will be smaller than when populations were stable and had a balanced structure of all its components. Therefore, in a reproductive population composed of mostly neophytes, the size of the females and the number of eggs laid will be smaller than those of an older, better-established, and reproductively mature population. Our data suggest that high recruitment rates are driving the observed slight reduction in body sizes in the southern Gulf of Mexico.

However, another explanation of the reduction in the body size is related to changes in the foraging habitats which have been caused by an ecological regime shift as a result of climate change. Size reduction has been detected in both green [42] and hawksbill turtles [29,43] in the West Atlantic and the Caribbean, and also in leatherback (*Dermochelys coriacea*) and loggerhead (*Caretta caretta*) turtles in other regions of the world, despite increasing trends in nest numbers [9], and this is attributed to the ecological changes of their foraging habitats. Although a reduction in the body size and being of an early age at maturity are expected in ectotherms as a result of an increase in the metabolic rate driven by higher temperatures [44], the reduction in other reproductive parameters, particularly the hatching and emergence success (see next section), suggest that the fitness of sea turtle populations could be at risk if temperatures continue to increase at the current speed. Sea turtles are resilient species and have gone through many climate change events in the past, but none of them occurred at the pace of the current warming period, and this can hinder their resilience.

### 4.3. Clutch Size, Hatching, and Emergence Success

The mean clutch size of the hawksbill turtles we recorded was lower than that reported for the same nesting sites in previous studies and other nesting sites in the Caribbean [11,45]. In contrast, the mean clutch size of our green turtles was similar to those in other nesting sites in the Caribbean and Atlantic region [46], except for Cayos Arcas and Isla Aguada. Although no significant trend was found in our data, the mean clutch size shows a decreasing pattern in our region that requires further attention, especially when this decrease has been detected at other hawksbill nesting beaches in the Caribbean region [29]. On the positive side, this could be related to the contribution of neophytes to the new population structure, and an effect before the stabilization stage. However, in sea turtles, studies have found a positive correlation between the clutch size, the size of females [47], and the quality of food resources [48,49]. Therefore, it could be a feasible hypothesis that under the continued decline of ocean productivity caused by the combined effect of global warming [50,51] and environmental pollution [52], the clutch size is being negatively affected. When the quality and amount of food are decreasing, the reproductive output is reduced. Unsuitable conditions on the foraging grounds partially explain the decreasing trend of the hatching success observed in this study, since these indirectly affect the fitness of the embryos [49]. This could be further escalated if the incubation temperatures go beyond suboptimal thresholds that will increase the mortality of the embryos [53].

Under natural conditions, more than 65% of the eggs laid by the females will produce hatchlings [54]. However, small variations in the incubation temperature can alter this percentage. The incubation temperature is influenced by several conditions, including the depth of the nest and the orientation of the beach. These variables affect the amount of heat that surrounds the nest, causing a variation in the sex [55], and the mortality of the embryos and hatchlings. Overall, temperatures above 30 °C will decrease the hatching success by about 25%, and an above 35 °C hatching success is reduced to zero [54]. In our study sites, the mean hatching and emergence success of hawksbill and green turtle undisturbed nests remains high (above 75%) in four of the seven nesting beaches because we assume that the microhabitat conditions of the nests, including the incubation temperature, remain within the optimal range. In the case of hawksbill turtles, the lethal temperatures that can cause the death of the embryos are dampened by the dune vegetation of the nesting beaches [56]. The low hatching and emergence success in San Lorenzo is caused by the relocation of nests to hatcheries. This nesting beach is 1.8 km long and is surrounded by rocks and summer houses; therefore, leaving nests in situ is not an option. On the other hand, Cayos Arcas is highly exposed to climatic events and extreme temperatures, whereas Isla Aguada has a lower beach quality caused by high erosion rates.

Between 2014 and 2020, the mean sand temperature was 29.3 °C in Celestún and 27.8 °C in El Cuyo and Holbox [57], suggesting a thermal gradient with higher to lower temperatures from the northeast to the northwest coast of the peninsula. However, some years presented mean temperatures above 31°C during the warmer months of the season. At Lechuguillas, the mean incubation temperature from 1997 to 2011 was 31.13 °C and it decreased to 28.4 °C between 2012 and 2018, with some days recording maximum temperatures of 35.8 °C. If the increase in the annual mean global temperature continues, the extreme variation can affect the sex ratio of the embryos at these nesting sites, favoring the production of females [58], but more importantly, more suboptimal conditions will be present and the hatching and emergence success will decline as a consequence of the increased mortality.

Putting this into perspective, even though management strategies such as the relocation of nests to shaded areas or incubation facilities to maintain hatching and emergence success is feasible if young turtles do not have healthy foraging areas to go to, all the effort invested at the nesting beaches will be in vain. It will be necessary to detect population changes at the foraging grounds caused by the reduction in the hatching and emergence success. Therefore, a more integrative approach to redirect the monitoring efforts for evaluating the climate change impacts on the critical developmental habitats for sea turtles is desirable and it requires coordinated actions of all the parties involved.

Previous studies using satellite telemetry to track the movements of post-nesting females [59,60,61] showed that hawksbill females nesting at the western and northern coasts of the Yucatán peninsula have different feeding grounds in this region, and even out of the Gulf of México. On the other hand, green turtles from different rookeries are more prone to aggregate in common feeding grounds mainly at the northeastern and northwestern corners of the Yucatán peninsula. These migratory patterns, together with the assessment of the reproductive parameters presented in this study, underline research gaps and opportunities to increase the biological knowledge of these syntopic species through an integrative approach.

## 5. Conclusions

The long-term monitoring and conservation actions conducted in the Southern Gulf of Mexico to recover hawksbill and green turtle populations show positive trends in the nest abundance in almost all of the nesting sites analyzed and provide useful information on the reproductive parameters that are necessary to evaluate population changes at a regional scale. Although the nesting trends of hawksbill and green turtles are slowly increasing, the reduction in female size and reproductive output (clutch size, hatching, and emergence success) in the last decade, while not statistically significant, is of a major concern as it suggests that other factors such as climate change and the associated ecological regime shift are affecting their recovery [54,62]. The reduction in the ocean productivity caused by warming seas [49] affects the quality of sea turtle foraging areas by reducing the availability and quantity of food [63], which in turn affects their reproductive output [46,47]. Therefore, monitoring and determining the conditions of the critical foraging areas of hawksbill and green turtles nesting in the southern Gulf of Mexico will help establish the bases for the restoration of these habitats, and aid in the recovery of the sea turtle population.

Our study emphasizes the need for an integrative approach to sustain the recovery of sea turtle populations. At present, the main threats to the nesting sites in the region that compete and oppose their recovery are coastal development, erosion, and plastic pollution, which require more effective actions to mitigate them and restore the nesting habitats. Minimizing threats and restoring critical foraging areas will require a characterization through comprehensive spatial analyses to determine what drivers affect these habitats and develop solutions to mitigate them. Given the migratory nature of sea turtles, these analyses will require sound and coordinated international collaborations between the different governments hosting these enigmatic species.

## Figures and Tables

**Table 1:** The average number of hawksbill (*Eretmochelys imbricata*) and green (*Chelonia mydas*) sea turtle nests laid annually (from 1990 to 2021) at seven nesting beaches in the Gulf of Mexico.

Nesting Beach	Hawksbill	Green
	Number of Nests	Number of Nests
	(Mean ± S. D.)	(Mean ± S. D.)
	(Range)	(Range)
Cayos Arcas	---No nesting records	369.76 ± 240.84
		(64–956)
Celestún	246.22 ± 124.92	1.41 ± 3.78
	(38–526)	(0–16)
El Cuyo	405.56 ± 183.49	1134.41 ± 1629.78
	(67–818)	(31–6679)
Hobox	514.84 ± 302.11	202.50 ± 238.01
	(67–1409)	(4–915)
Isla Aguada	250.41 ± 120	1400.31 ± 1747.01
	(124–655)	(38–5974)
Lechuguillas	1.7 ± 1.16	2460.29 ± 2888.12
	(0–4)	(61–11,044)
San Lorenzo	85.89 ± 72.52	No nesting records
	(19–365)	

**Table 2:** Results of the generalized additive models to evaluate the temporal effects of the nesting season on the number of nests of hawksbill (*Eretmochelys imbricata*) sea turtles at five nesting beaches in the Gulf of Mexico. Lechuguillas was excluded from the analysis due to its small sample size, and Cayos Arcas is not a Hawksbill nesting site.

N Nest as Function of s (Season, by = Beach) + Beach (Negative Binomial)				
Hawksbill Turtle				
	Estimate	Std Error	z Value	Pr (>|t|)
Intercept	5.444081	0.05783	94.081	<2 × 10^−16^ ***
El Cuyo	0.50269	0.08143	6.173	6.69 × 10^−10^ ***
Holbox	0.68002	0.08138	8.356	<2 × 10^−16^ ***
Isla Aguada	0.03654	0.08172	0.447	0.655
San Lorenzo	−1.07578	0.09144	−11.764	<2 × 10^−16^ ***
R-sq (adj) = 0.782	Deviance explained = 85.2%	REML = 942	Scale est. = 1	n = 156

The significance codes of the test statistics are: 0 ‘***’, 0.001 ‘**’, 0.01 ‘*’, 0.05 ‘.’, and 0.1 ‘ ’ 1.

**Table 3:** Results of the Generalized Additive Models to evaluate the temporal effects of the nesting season on the number of nests of green (*Chelonia mydas*) sea turtles at five nesting beaches in the Gulf of Mexico. San Lorenzo and Celestún were excluded from the analysis due to their small sample size.

N Nest as Function of s (Season, by = Beach) + Beach (Negative Binomial, Link = Log)				
*Chelonia mydas*				
	Estimate	Std Error	t Value	Pr (>|t|)
Cayo Arcas	5.4049	0.2735	19.762	<2 × 10^−16^ ***
El Cuyo	1.0033	0.2995	3.350	0.000809 ***
Holbox	−0.5224	0.2999	−1.742	0.081529
Isla Aguada	1.2739	0.2995	4.253	2.11 × 10^− 5^ ***
Lechuguillas	1.9379	0.3040	6.374	1.84 × 10^−10^ ***
R-sq. (adj) = 0.651	Deviance explained = 79.05%	REML = 1011.5	Scale est. = 1	n = 141

The significance codes of the test statistics are: 0 ‘***’, 0.001 ‘**’, 0.01 ‘*’, 0.05 ‘.’, and 0.1 ‘ ’ 1.

**Table 4:** Summary statistics for morphological and reproductive indicators of hawksbill (*Eretmochelys imbricata*) and green (*Chelonia mydas*) turtles that nested at seven index beaches in the Gulf of Mexico from 1990 to 2021.

Nesting Site		Female Size (CCLmin, cm)	Clutch Size (Egg Number)	Hatching Success (%)	Emergence Success (%)	Study Period
**Hawksbill Turtle**						
Celestún	Mean	89.15	131.34	83.36	77.68	1995–2021
	S.D.	5.20	31.87	25.14	30.39	
	Range	70–110	33–193	0–100	0–100	
	N	1298	4236	4236	4236	
El Cuyo	Mean	90.01	136.69	80.98	74.24	1995–2021
	S.D.	5.10	32.84	25.92	31.75	
	Range	67–120	33–193	0–100	0–100	
	N	2734	5202	5202	5202	
Holbox	Mean	88.72	121.17	91.72	87.00	1990–2021
	S.D.	4.75	33.30	22.75	28.25	
	Range	67–109	33–193	0–100	0–100	
	N	1721	7436	7436	7436	
Isla Aguada	Mean	89.99	128.25	56.24	40.84	1998–2021
	S.D.	5.51	32.10	30.92	34.27	
	Range	69.6–113	34–193	0–100	0–100	
	N	1669	3270	3270	3270	
Lechuguillas	Mean	104	94.5	94.09	93.08	2018
	S.D.	4	6.36	2.64	1.21	
	Range	100–108	90–99	92.22–95.95	92.22–93.93	
	N	3	2	2	2	
San Lorenzo	Mean	84.63	146.05	61.59	58.37	2003–2016
	S.D.	5.15	30.48	23.33	22.84	
	Range	71.08–98	60–193	1.18–100	0–93.57	
	N	141	220	220	220	
**Green turtle**						
Cayos Arcas	Mean	103.19	116.64	71.56	58.29	2002–2014
	S.D.	5.17	20.62	28.26	34.80	
	Range	76.9–116.34	56–153	0–100	0–100	
	N	505	427	427	427	
Celestún	Mean	105.94	116.89	79.90	76.88	1995–2021
	S.D.	8.55	19.97	17.21	21.16	
	Range	90.76–115.35	71–152	7.84–100	3.92–100	
	N	6	19	19	19	
El Cuyo	Mean	103.72	110.27	89.40	86.43	1995–2021
	S.D.	6.25	19.81	16.71	20.80	
	Range	80.9–124.05	54–153	0–100	0–100	
	N	3386	5476	5476	5476	
Holbox	Mean	103.13	113.05	91.20	88.08	1990–2021
	S.D.	5.59	19.99	17.62	21.59	
	Range	85.85–124.21	54–153	0–100	0–100	
	N	532	1335	1335	1335	
Isla Aguada	Mean	105.66	106.52	65.31	52.26	1998–2021
	S.D.	5.38	20.05	26.01	33.77	
	Range	78.1–124	54–153	0–100	0–100	
	N	7184	12720	12720	12720	
Lechuguillas	Mean	103.95	101.81	84.09	81.19	1995–2021
	S.D.	5.45	18.54	19.81	22.65	
	Range	85–122.94	54–154	0–100	0–100	
	N	1746	26,110	26,110	26,110	

## Data Availability

Data used for this study are available upon request.

## Supplementary Materials

The following supporting information can be downloaded at: https://www.mdpi.com/article/10.3390/ani12233280/s1, Figure S1: Time series of the number of nests for hawksbill turtles (*Eretmochelys imbricata*) at Celestún (A), El Cuyo (B), Holbox (C), Isla Aguada (D), San Lorenzo (E), and Lechuguillas (F) from 1990 to 2021 Figure S2: Time series of the number of nests for green turtles (*Chelonia mydas*) at Lechuguillas (A), Isla Aguada (B), El Cuyo (C), Cayo Arcas (D), Holbox (E), and Celestún (F) from 1990 to 2021.
