# Supplementary material for: Trends in Reproductive Indicators of Green and Hawksbill Sea Turtles over a 30-Year Monitoring Period in the Southern Gulf of Mexico and Their Conservation Implications
Source: Animals (Basel). 2022 Nov 24;12(23):3280. doi: 10.3390/ani12233280 (PMC9739169; doi:10.3390/ani12233280)
Supplement: Supplementary file 1 [file animals-12-03280-s001.zip › animals-2017121-supplementary material.pdf]

animals
MDPI

Supplementary Material

Trends in reproductive indicators of green and hawksbill sea turtles over a 30-year monitoring period in the Southern Gulf of Mexico and their conservation implications

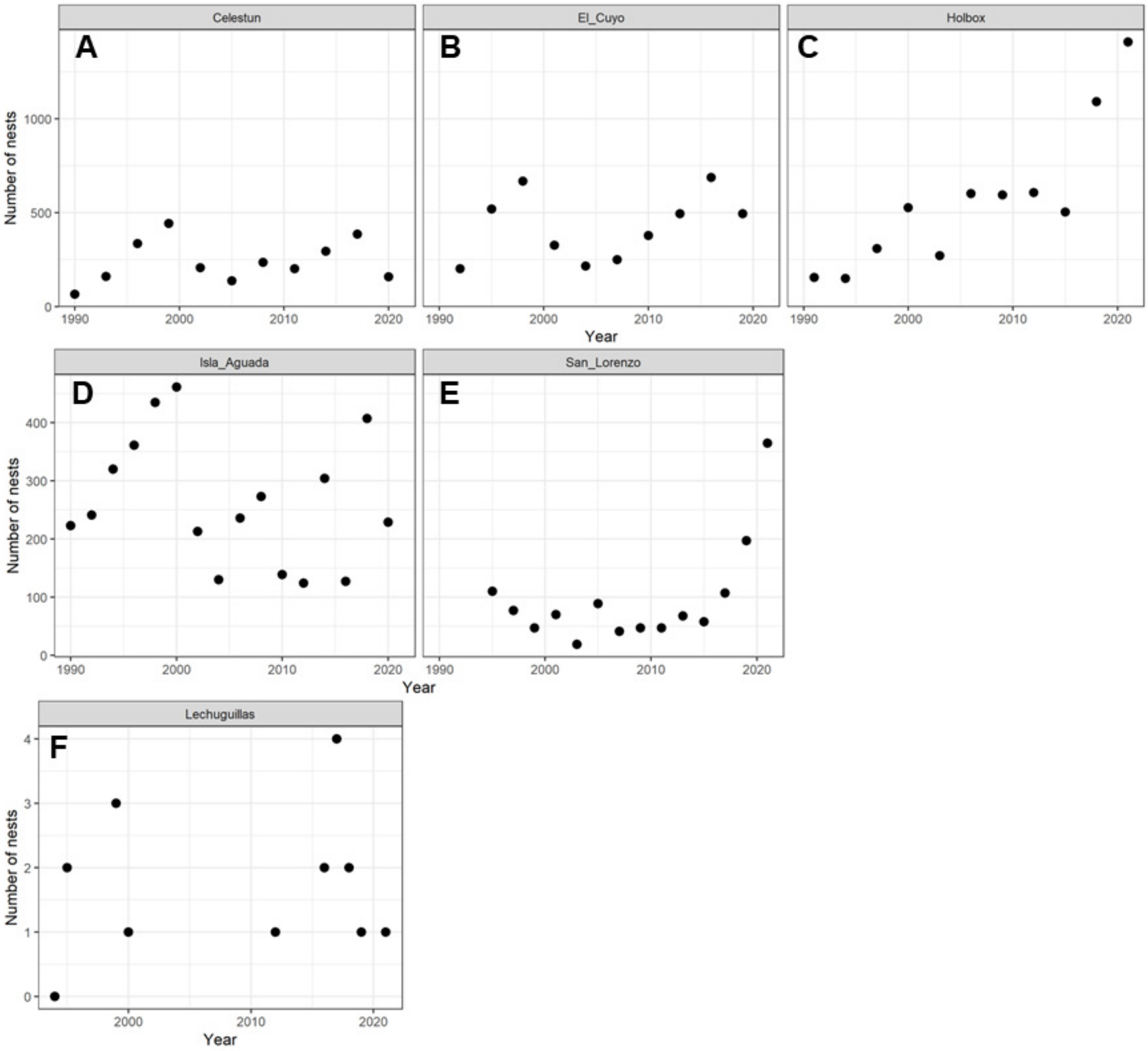

Figure S1. Time series of the number of nests for hawksbill turtles (*Eretmochelys imbricata*) at Celestún (A), El Cuyo (B), Holbox (C), Isla Aguada (D), San Lorenzo (E), and Lechuguillas (F) from 1990 to 2021. Panels are arranged in different levels based on the magnitude of nests abundance, Top (A, B, C): 0 to 1,500; Middle (D, E): 0 to 500; Bottom (F): 0 to 4.

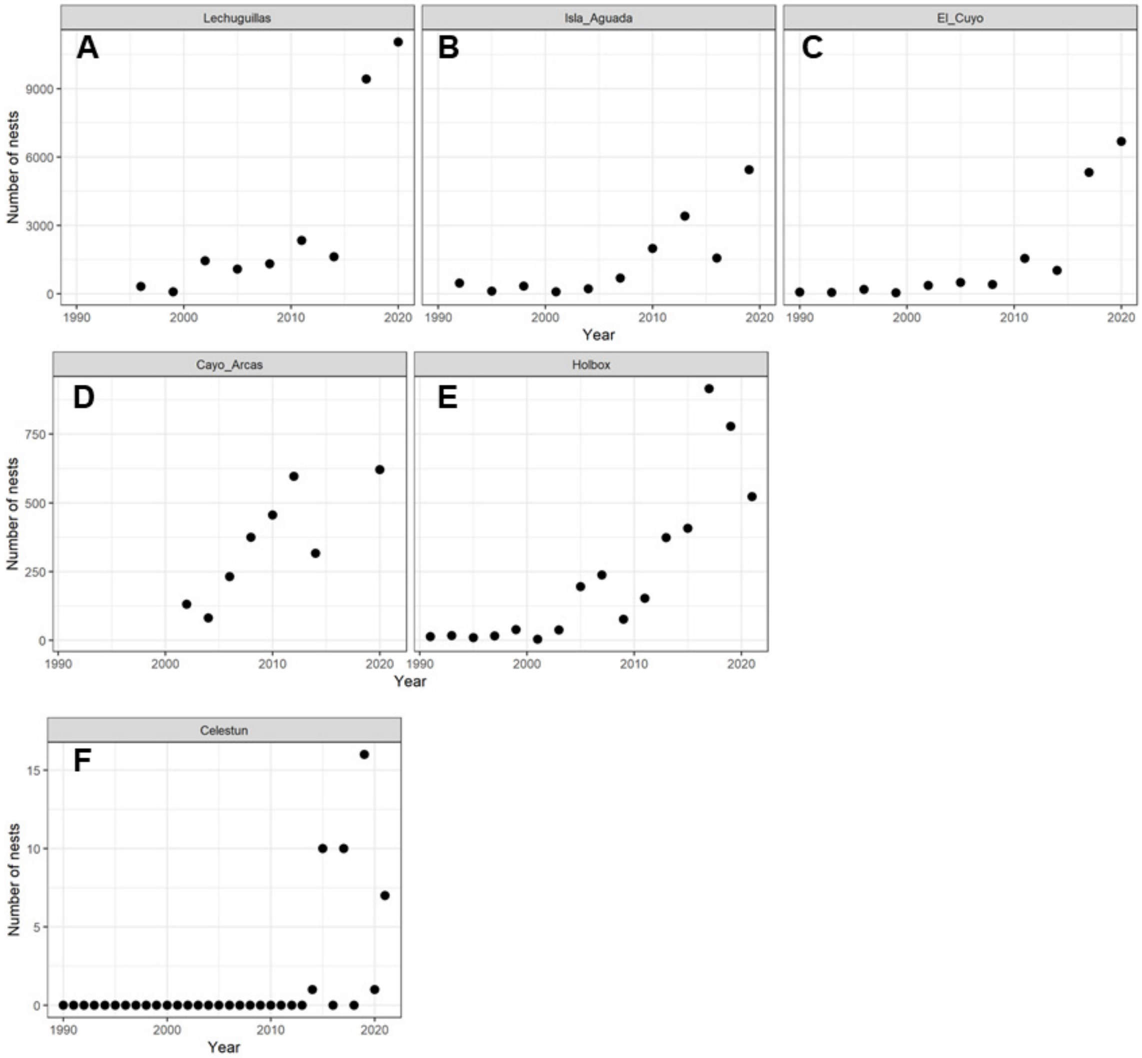

Figure S2. Time series of the number of nests for green turtles (*Chelonia mydas*) at Lechuguillas (A), Isla Aguada (B), El Cuyo (C), Cayo Arcas (D), Holbox (E), and Celestún (F) from 1990 to 2021. Panels are arranged in different levels based on the magnitude of nests abundance, Top (A, B, C): 0 to 11,000; Middle (D, E): 0 to 900; Bottom (F): 0 to 20.
